# Genetic Biofortification of Winter Wheat with Selenium (Se)

**DOI:** 10.3390/plants13131816

**Published:** 2024-07-01

**Authors:** Katarina Sunic, Valentina Spanic

**Affiliations:** Department for Cereal Breeding and Genetics, Agricultural Institute Osijek, Južno Predgrađe 17, 31000 Osijek, Croatia; katarina.sunic@poljinos.hr

**Keywords:** genetic biofortification, wheat, selenium

## Abstract

Wheat is one of the three most important cereals in the world, along with rice and maize. It serves as the primary food and source of energy for about 30–40% of the world’s population. However, the low levels of micronutrients in wheat grains can lead to deficiencies of those micronutrients in people whose dietary habits are mostly based on cereals such as wheat. Apart from iron (Fe) and zinc (Zn), a lack of selenium (Se) is also one of the biggest problems in the world. The essentiality of Se has been confirmed for all animals and humans, and the lack of this micronutrient can cause serious health issues. Wheat dominates the world’s cereal production, so it is one of the best plants for biofortification. Due to the fact that agronomic biofortification is not an economical or environmentally acceptable approach, genetic improvement of cereals such as wheat for the enhanced content of micronutrients in the grain represents the most efficient biofortification approach.

## 1. Introduction

Winter wheat (*Triticum aestivum* L.) is an allohexaploid species that was formed through hybridization approximately 8000 to 10,000 years ago, and, since then, its germplasm has continued to evolve in parallel with ancient human migration routes [1]. Today, with more than 220 million hectares planted, wheat is one of the three most important cereals in the world, together with rice and maize, and represents the main source of food and energy for about 30–40% of the world’s population, especially in developing countries [1,2,3,4]. Among these, hexaploid wheat accounts for 95% of all wheat species grown globally, while the remaining 5% is durum wheat (*Triticum turgidum* ssp. *durum*) [4]. According to the available data, the biggest wheat producers in the world are China and the European Union, and the amount of wheat produced by them provides more than 20% of the world’s food supply [5]. It is predicted that, by 2050, the need for wheat production will increase due to the increase in the world population, accelerated urbanization, and industrial development, which at some point could result in a shortage of food on the world market [6]. Although an increase in wheat production is predicted in the coming years, the increase in its nutritional quality during production falls mostly into the background. With the continuous increase in the human population, the need for food also grows, and the lack of suitable agricultural and arable land leads to intensive exploitation of natural resources and often products of low and insufficient quality.

In addition to insufficient wheat production, low levels of micronutrients in wheat grains can lead to micronutrient deficiency in people whose eating habits mostly rely on cereals such as wheat [2]. Although approximately 49 micronutrients are considered essential for the normal functioning of human metabolism, the lack of any of them significantly impairs health [7]. It can negatively affect growth, cognitive development, and the functioning of the immune system, often resulting in lifelong consequences [8]. Although significant improvements have been made in recent years, the growing human population continues to lead to a certain increase in malnutrition, primarily in highly populated areas of the world [9]. Therefore, malnutrition, or “hidden hunger”, as it is often called today, represents a significant obstacle to human health and economic growth, but also undermines the efforts made to reduce poverty [10]. Looking at the global level, more than two billion people are affected by micronutrient deficiency, where one of the biggest problems is iron (Fe) and zinc (Zn) deficiency [11]. Apart from Fe and Zn, selenium (Se) deficiency also represents a problem due to the fact that Se is an essential micronutrient for humans and animals [12]. Wheat is one of the main food resources worldwide. Therefore, a sufficient Se content in wheat grains can maintain the well-balanced Se status of the world population. In this article, we reviewed the role of Se and different strategies that can be used to improve Se content in wheat through biofortification, with a highlight on genetic biofortification.

## 2. Selenium and Its Role in Human Health

**Figure 1:**
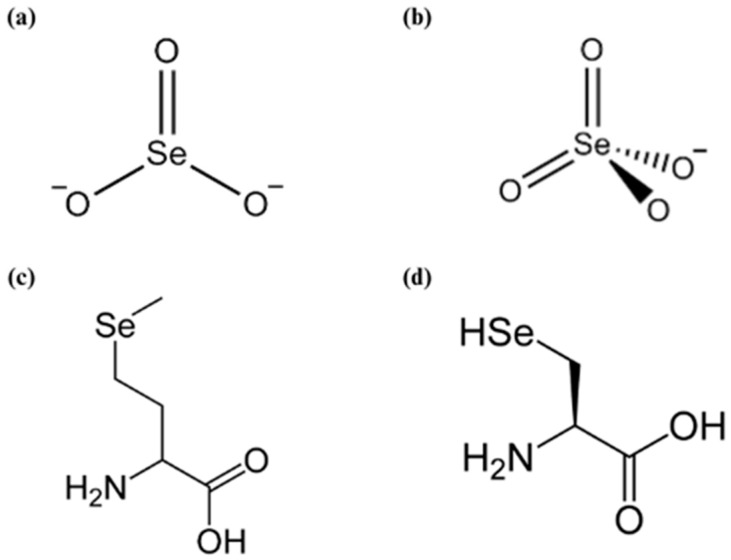
Structural formulas of (**a**) selenite, (**b**) selenate, (**c**) selenomethionine, and (**d**) selenocysteine.

Historically, research on the effect of Se on human health was primarily focused on the possible harm caused by excessive Se doses in humans [13]. The essentiality of this trace element was confirmed in the middle of the last century and until now there has been a growing interest in Se research [14]. The presence of Se in soil varies worldwide. On a global scale, the total amounts of Se in soil usually fluctuate between 0.01 and 2.0 mg kg^−1^ with an average value of 0.33 mg kg^−1^ [15,16]. The highest presence of Se in the soil (up to 1200 mg kg^−1^) is found in soils derived from seleniferous parent materials which prevail in the parts of United States of America, Canada, South America, China, Russia, and Australia, while the rest of the world is mostly poor in this element [17,18]. Nevertheless, substantial amounts of Se are introduced into soils via the deposition of Se from both natural and anthropogenic sources [19]. Se occurs in the soil in several inorganic forms, such as elemental Se (Se^0^), selenide (Se^2−^), selenite (SeO_3_^2−^), and selenate (SeO_4_^2−^) (Figure 1) [20].

In soil, inorganic Se exists in three phases: fixed, adsorbed, and soluble and it is thought that only the adsorbed and soluble forms of Se are accessible for plant absorption [13]. Furthermore, the presence of selenate (+6 oxidation state) and selenite (+4) forms in plants shows significant variation, with selenate being absorbed at a much faster rate than selenite in the majority of soil conditions [13]. The rate at which Se is absorbed by plants is very important, and depends of plant type, pH level, soil composition, rainfall distribution, microbial activity, and other influences [21]. Due to a lack of Se in the soils of specific regions, inadequate dietary intake might occur [22].

Food is the main source of Se in the human body, and its content in food of plant origin is most often a reflection of its presence in the soil in the area of plant cultivation [23]. As for plant-based foods, Brazil nuts are considered to have the highest Se content (up to 512 μg g^−1^) [24], while cereals and cereal products are the main source of Se in countries around the world. Except for cereals and other plant sources of Se, the main food groups rich in Se are meat, fish, eggs, milk, and dairy products [13]. Along with rice, wheat dominates the world’s cereal production, and for this reason, it is one of the best plants for biofortification [15]. Ever since Se was recognized as an essential micronutrient, a large number of different studies have described its significant impact on human health [25]. According to the recommended dietary allowance (RDA), the recommended average daily level of Se intake for humans from 14 years old is 55 μg. During pregnancy or lactation, the daily level of Se intake should be slightly increased (60 and 70 μg, respectively) (Table 1).

The range of Se consumption that may lead to either deficiency or toxicity is quite small and the health consequences are determined by the degree of exposure and the individual’s Se status [13]. Also, attention should be paid to people who have gastrointestinal health problems and food sensitivities that limit the availability of Se from food sources such as cereals. Despite a large number of studies on Se deficiency, there is limited evidence regarding the extent of exposure and the particular health consequences influenced by Se exposure [26]. Although there is a lack of consensus regarding the safe range of exposure, recent estimates indicate that intakes above 900 μg day^−1^ may be harmful while those below 30 μg day^−1^ are insufficient [13]. The highest levels of Se intake are documented in parts of China and Venezuela [27], while for most of the European countries with accessible estimates the average daily intakes are less than 50 µg per person which is near or below recommended intake level [28].

As a component of the amino acid selenocysteine (SeCys) in the enzyme’s active sites, Se participates in numerous metabolic processes in humans (Figure 1) [29], but the main functions in which Se plays a vital role is thyroid gland functioning and thyroid hormone biosynthesis and metabolism, the antioxidative defense system and oxidative metabolism, and the immune system [13]. The redox-protective functions of selenoproteins are especially significant in the thyroid gland, whose cells produce H_2_O_2_ (as well as reactive oxygen species) that are necessary for the production of thyroid hormones. Besides that, even more relevant is the direct involvement of selenoenzymes (iodothyronine deiodinases) in thyroid hormone metabolism [30]. Furthermore, four significant selenoproteins, glutathione peroxidases (GPxs), including cytosolic GPx, gastrointestinal-specific GPx, plasma GPx, and phospholipid hydroperoxide GPx, are well-characterized major enzymes of the human antioxidant defense systems [13]. In the immune system, Se stimulates the activity of immune cells such as helper T, cytotoxic T, and natural killer (NK) cells [31]. Se deficiency leads to a number of serious diseases such as Keshan and Kashin–Beck disorders. Moreover, a lack of Se is linked to muscle tissue death, hypothyroidism, cardio-cerebrovascular disease, male infertility, a higher occurrence of many types of cancer, and a weakened immune system [15,32]. As Se deficiency is associated with autoimmune disorders, it has also been recognized as a risk factor for Hashimoto’s thyroiditis [33]. Anxiety and depression, which are also associated with Se insufficiency, exhibit similar symptoms to Hashimoto’s disease [34]. All the mentioned disorders occur as a result of changes in the immune system associated with Se deficiency, such as suppression of the immune response to various viral and bacterial infections, but also a decrease in the activity of lymphocytes and macrophages involved in various immune processes [32]. Although much less common, as mentioned before, excessive intake of Se can also be harmful to health [35]. The symptoms can include garlicky breath, dermatitis, hair and fingernail loss, acute respiratory distress, myocardial infarction, and kidney failure [29].

## 3. Selenium in Plants

**Figure 2:**
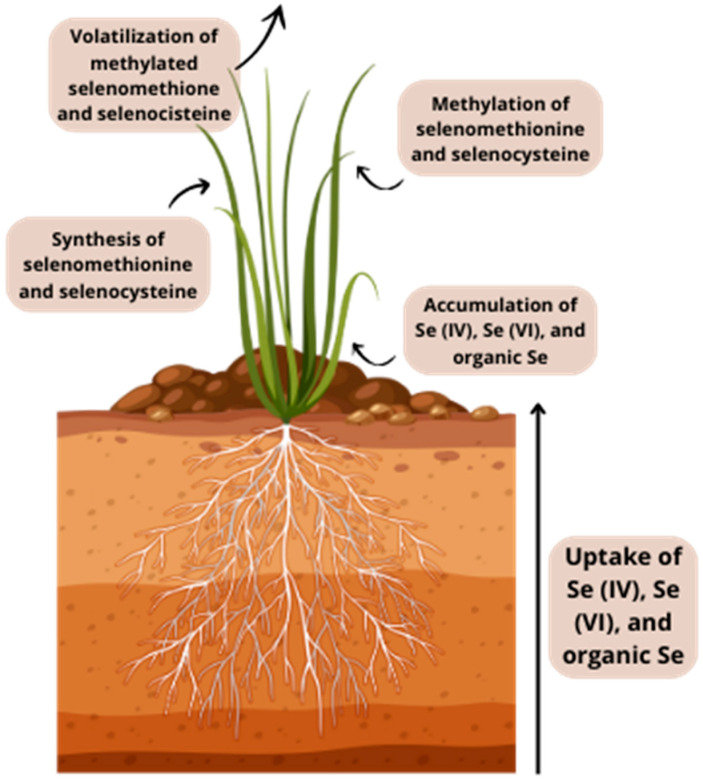
Illustration showing simplified uptake of selenium by plants.

Plants absorb, transport, and incorporate Se into their system via the sulfur (S) pathway due to their chemical similarities [36]. Sulfate transporters SULTR1 and SULTR2 play a crucial role in the uptake of selenate, while selenite is mostly taken up through phosphate transporters [37]. Besides the aforementioned transporters, aquaporins and silicon influx transporters have also been found to play a role in the transportation of selenite [38]. Organic forms of Se, SeCys and selenomethionine (SeMet) (Figure 1), are taken up by plants through amino acid permeases [39]. Following uptake by the root cells, selenate is transformed into adenosine 5′-phosphoselenate (APSe) and subsequently reduced to selenite through the action of adenosine 5′-phosphosulfate reductase (APR) [40]. Sulfite reductase (SiR) located in chloroplasts converts selenite into selenide, which can then be further converted to SeCys by cysteine synthase. SeCys can also be transformed into different selenoproteins or converted into other chemical compounds. The compounds mentioned are SeMet, methylselenocysteine (MeSeCys), methylselenomethionine (MeSeMet), and elemental Se (Se^0^) [41,42]. Due to the inability of plants to differentiate between SeCys and Cys, the inclusion of SeCys in proteins results in protein malfunction and subsequently causes toxicity in plants [36]. The transformation of SeCys into different chemical compounds is considered to be a detoxifying process, as it hinders its integration into proteins [43]. SeMet and SeMeCys can undergo additional transformations to produce volatile selenide compounds, such as dimethylselenide (DMeSe) or dimethyldiselenide (DMeDSe) [36] (Figure 2).

The absorption of Se from soils into plants is influenced by various factors, such as soil properties, bioavailability and Se speciation, the presence of other competing ions, and the specific plant species [42]. Selenate is considered to be the most accessible form for plants, whereas selenite is considered to be less accessible due to its higher tendency for adsorption onto soil particles. Unlike selenate, the majority of selenite is retained in the root since it undergoes rapid conversion into organic compounds [42,44]. On the other hand, the rate of Se uptake for organic species is significantly higher compared to selenate, while selenate exhibits greater mobility within the plant [42]. Additional organic species such as selenocystathione, glutamyl-methylselenocysteine, and various selenoproteins can be identified as metabolic intermediates in plants [45,46]. Se-containing proteins contain Se-amino acids in their peptide chains, which are believed to be incorporated into peptide synthesis via the metabolic pathway of their sulfur analogs. The four primary categories of plant proteins containing Se are albumin, glutelin, globulin, and prolamin [47]. The actual absorption of Se by the mammalian metabolism is dependent on its speciation and estimating the benefits of Se from diet requires not only considering the amount of Se present, but also having precise information about the Se species [42,48].

**Figure 3:**
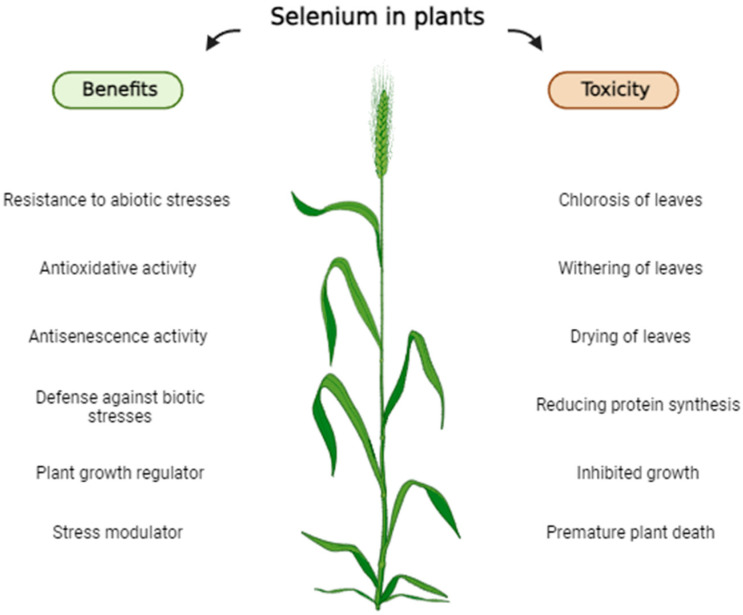
Beneficial and harmful effects of selenium in plants.

Although the essentiality of Se for plants is still undetermined, some studies have shown a beneficial effect of Se on increasing crop productivity (Figure 3) and it can be utilized to develop tolerance against other abiotic stresses such as high temperature, freezing, drought, and salinity [32]. Selenoproteins have a role as antioxidants in plant metabolism through the glutathione peroxidase pathway, and provide an increased activity for enzymatic and non-enzymatic compounds which reduce reactive oxygen species (ROS) accumulation and thus oxidative stress [49]. For example, treatments at 1.0, 2.0, and 3.0 mg kg^−1^ of Se significantly increased root activity, proline content, peroxidase (POD), and catalase (CAT) activities, carotenoids (Car) content, and chlorophyll content, and reduced the malondialdehyde (MDA) content of wheat seedlings [50].

In other research, the increasing content of anthocyanin, sugars, and proline with the application of Se in adequate dosages may suggest the interference of non-enzymatic substance synthesis [51]. The photosystem II (PSII) has also shown a rise in chlorophyll concentration and photosynthetic quantum performance activities. It might be evident that Se is a vital element in several physiological and biochemical processes. It was also reported that Se treatment at a low concentration exerts positive effects on plant growth, development, and grain yield [52]. Furthermore, Se also shows antioxidant properties which promote heavy metal detoxification by alleviating physiological stress [36,53]. This is especially important because heavy metals can be one of the major constraints in agricultural productivity [54]. Since heavy metals may inhibit photosynthesis by decreasing chlorophyll contents [55], Se stimulates respiration rates and the flow of electrons in the respiratory chain and accelerates chlorophyll biosynthesis [56].

Besides tolerance for heavy metal toxicity, it was reported that Se and Se nanoparticles (SeNPs) can also protect plants from biotic stresses. In the research of Shahbaz et al. [57], spot blotch disease-stressed wheat plants responded favorably to 30 µg mL^−1^ foliar treatment of SeNPs, both physiologically and biochemically. Plant morphological, physiological, and biochemical characteristics were thereby enhanced due to the activation and synthesis of plant defense-related enzymatic and non-enzymatic compounds such as proline, phenols, and flavonoids. This is especially important with the today’s global climate change, when Se may be used to enhance crops’ tolerance to abiotic and biotic stresses.

However, high doses of Se in plants lead to selenium toxicity or selenosis which occurs in plants when the content of Se surpasses the optimal level (Figure 3). Se induces toxicity through two mechanisms: the formation of defective selenoproteins and the induction of oxidative stress [58]. Malformed selenoproteins occur as the consequence of the erroneous substitution of SeCys or SeMet for cysteine (Cys) or methionine (Met) within the protein chain. Substituting SeMet with SeCys results in higher reactivity and significantly impairs protein functioning [59]. On the other hand, at elevated concentrations, Se functions as a pro-oxidant and produces reactive oxygen species, leading to oxidative stress in plants and to a reduction in the concentration of glutathione (GSH) [60].

## 4. Biofortification of Wheat with Selenium

Various agricultural measures to improve the nutritional value of food crops could help improve human nutrition, primarily in less developed countries [10]. Micronutrients in the diet can be increased by various supplements, industrial fortification, and biofortification [2], and it is biofortification that is gaining more and more importance when it comes to improving the nutritional quality of food [61]. Biofortification or biological fortification refers to the process of increasing the bioavailable concentrations of micronutrients in the edible parts of crops using agronomic interventions or genetic selection [62]. It is ranked as the fifth-best and most cost-effective solution for reducing the global problem of malnutrition or hidden hunger [3]. The content of micronutrients in cereal grains can be increased by agronomic and genetic biofortification [63]. However, there are many challenges when talking about Se biofortification and different factors can affect Se content, forms, and bioavailability in biofortified wheat. Many soil characteristics and conditions influence Se biofortification. Certain research indicates problems with Se biofortification through soil due to the reduced absorption by plants and the potential cost drawbacks connected with this approach [64]. Furthermore, soil ions can interfere with the absorption of Se by plants. It has been observed that the addition of S to the soil can hinder the uptake of Se by plants, as they both utilize the same metabolic pathway for translocation [65]. In clayey soils, Se has low bioavailability due to its affinity for clay minerals [66]. The adsorption of Se is also influenced by soil pH. Selenite exhibits low solubility in acidic and neutral soils, but selenate is more easily taken up by plants in neutral and alkaline soils [67]. In addition, the concentration of Se in food is influenced by cooking and preservation processes. The process of flour milling results in a reduction in Se in wheat, leading to losses. The amount of Se lost in white all-purpose flour was 12–68% greater than in whole wheat flour, while the bioaccessibility of Se in whole wheat and white all-purpose flour, treated with different levels of Se, varied from 6% to 38%. The authors concluded that white all-purpose flour exhibited greater bioaccessibility of Se compared to whole wheat flour. In a study conducted by Reeves et al. [68], it was discovered that the bioaccessibility of Se in refined wheat flour (mostly the endosperm), wheat shorts (which predominantly consist of the germ), and wheat bran were 100%, 85%, and 60%, respectively. The limited bioavailability of Se in bran is mostly due to the fact that the selenoprotein is enveloped by the indigestible fiber present in this component. Furthermore, different forms of exogenously applied S can have varying impacts on the bioaccessibility of Se in wheat. Although Se is an essential micronutrient, its biofortification is hindered by its toxicity beyond a specific threshold, as the range between biofortification and toxicity is small. Animal Se supplementation can lead to toxic or potentially fatal consequences while in non-accumulator plants, such as wheat, it can have a negative impact on plant physiology, growth, and development [69].

### 4.1. Agronomic Biofortification

Agronomic biofortification is a method based on the use of fertilizers in the soil (basal application) or foliar application on plants [70]. Agronomic soil biofortification is a simple but mostly short-term solution important to complement genetic biofortification, especially when the soil in the target region is limited to a certain level of micronutrients available for use [70,71]. Enhancing the Se content of essential crops like wheat through fertilization is an effective method to augment Se levels in the human diet, hence mitigating Se deficiency. In biofortification research, Se fertilizers are commonly applied at low rates of 10–20 g Se ha^−1^. In order to facilitate the use of a small quantity of Se in the fields, it is commonly combined with other fertilizer matrices. These matrices, which are commonly known as “carriers” of Se, typically provide a combination of nutrients or primarily macronutrients, such as urea and calcium nitrate [72]. Furthermore, the form in which Se is applied affects its effectiveness for biofortification [73].

In addition to research on the topic of which form of Se is more effective for biofortification, many studies have also dealt with the problem of which method of agronomic biofortification is more effective: basal application in the soil or foliar application on plants. The reason why many studies recommend the latter application during the growing season probably lies in the fact that Se has more rapid uptake and assimilation at this time due to application at a later growth stage of the plant, less influence of root-to-shoot ratio on translocation to the edible plant parts, and the avoidance of losses through fixation in soils [72]. Furthermore, selenite in the soil is an oxyanion and due to its mobility can be harmful to the environment because of its toxicity to animals [74]. Other studies have also reported about excessive oxyanions leaching from soils into water and causing severe environmental problems [75]. For these reasons, agronomic biofortification is not an economically or environmentally acceptable approach because only a small part of the applied micronutrient is available to plants for uptake. However, according to recent reports, introducing rhizospheric or endophytic microorganisms to soil or crops might increase the levels of micronutrients in different parts of plants, making them a “greener” alternative for sustainable agriculture, as they utilize naturally occurring biological processes [76,77]. Microbe-mediated biofortification can work in three different ways: (i) it can improve nutrient levels in plants by improving the availability of nutrients, (ii) it can directly synthesize and release micronutrients, and (iii) it can induce plants to synthesize micronutrients [76]. Endophytes and mycorrhizal fungi found in plant roots in these ways can, e.g., enhance the process of phosphate solubilization, the creation of siderophores for Fe chelation, and the release of enzymes and organic acids. These activities contribute to the increased movement of minerals from the soil to the aboveground sections of the plant [78]. Durán et al. [79,80] in their studies reported enhanced Se content in wheat grain in plants co-inoculated with a mixture of selenobacteria and arbuscular mycorrhizal fungi in comparison to plants without any mycorrhizal association and suggested certain endophytic strains as a microbial inoculant for Se biofortification. Along with microbe-mediated biofortification, genetic improvement of cereals for micronutrient content in grains is also one of the most effective biofortification approaches [61,81,82].

### 4.2. Genetic Biofortification

Since the transition from hunter–gatherer to sedentary lifestyles and the development of agriculture, wheat has been essential for the development of many civilizations. It is constantly shaped by selection to meet human needs and to adapt to different environments [1]. The genetic biofortification of wheat implies wheat breeding and genetic engineering. Both approaches are often compared because, compared to agronomic biofortification, both involve changing the genotype of targeted plants [83]. When comparing plant breeding and genetic engineering, breeding is still more practical and in much wider use compared to genetic engineering in wheat biofortification due to the costs of creating genetically modified organisms and the problems associated with placing such products on the market [84].

Biofortification by wheat breeding is achieved when the genetic variability of wheat is easily accessible from its primary, secondary, or tertiary gene pool, and a large number of still insufficiently researched wild relatives could significantly contribute to its genetic improvement [85]. Although wheat breeding is generally considered a better method of biofortification, conventional breeding also has certain drawbacks that could primarily be reflected in the high costs of measuring the micronutrient content required with the traditional breeding approach based on phenotyping [2].

Marker-assisted selection (MAS) and other marker-based approaches, such as marker-assisted recurrent selection (MARS) and genomic selection (GS), could therefore represent somewhat more efficient approaches to increase micronutrients through breeding [61]. Furthermore, very little attention has been given to new discoveries related to wheat genomics and how precisely they can be applied in wheat biofortification [2]. In order to be able to breed wheat for biofortification with the help of MAS, it is important to have information about the genomic regions that control the content/concentration of certain micronutrients. For this purpose, numerous studies are being carried out that enable the identification of a large number of different quantitative trait loci (QTLs) and genes that affect micronutrient contents [61].

However, when compared to MAS, GS has certain advantages for the purpose of biofortification. GS can capture a much larger extent of genetic variation for a particular trait that has been selected and this is due to the inclusion of all markers (with smaller and larger effects) during the genomic estimation of breeding value (GEBV) [86]. GS can significantly reduce the cost and time of wheat breeding [82].

#### 4.2.1. Breeding Wheat for Increased Uptake and Accumulation of Selenium

It has been proven that the increase in wheat grain yield in recent years has resulted in a decrease in the content of Se in the wheat grain [87], which indicates an increased need for selection of wheat genotypes with improved Se intake [61]. Biofortification for Se using breeding is probably one of the most established, sustainable, and long-term methods for the selection process of crops such as wheat [8]. The goal of this type of genetic biofortification is most often to create genotypes with a moderate to high capacity for uptake and translocation of Se to the edible parts of the plant or genotypes with a preferred uptake of organic forms of Se such as SeMet and/or methyl-selenocysteine (MetSeCys) [35,88].

Se in wheat grain is one of the most biologically available forms of Se. But despite this fact, wheat has very low concentrations of micronutrients in relation to human daily needs. Although considerable effort has been devoted to the study of genetic variation in Se content in wheat grain, there are many more studies and research on, for example, genetic variation in Zn and Fe content. In cultivated wheat, the variation in the Se concentration in the grain is relatively small, and, by all accounts, it seems that there is not much space for genetic improvement of this trait [89]. However, certain wild relatives of wheat, such as wild emmer wheat (*Triticum turgidum* ssp. *dicoccoides*), the tetraploid ancestor of today’s cultivated durum wheat and bread wheat, possess a wide allelic variation that is significant for the improvement of various economically important traits in cultivated wheat [90]. In the research by Yan [91], high concentrations of Se and significant variations in Se content among genotypes of wild two-grain spelt were found, and the variations and absolute values of Se concentration were much higher than those found between genotypes of hexaploid and other tetraploid wheat.

Se can be introduced into plants as selenate, selenite, or in an organic form, and it is considered that it is redistributed in plants in the form of selenite or the organic form via the phloem. Most of the genetic variation in Se content in the shoot is related to the condition of Se deficiency, while no genetic variation was observed in the condition of sufficient amounts of Se [92]. Such findings suggest that Se concentrations in the shoot could be evolutionarily limited [93]. Similar results have been obtained in other studies, and they point to the existence of a genetic component that controls the intake of Se in conditions of its reduced presence in the soil. Such results would mean that, if Se is present in the soil in insufficient amounts, the presence of large concentrations of Se in the grain is determined by the genotype. There is a strong positive correlation between the ability of a wheat genotype to accumulate Se and the concentration of Se in the grain [92]. Different studies have rather conflicting results when it comes to genetic variability among wheat genotypes for Se content in wheat grain. Thus, some studies have determined that there is no genetic variability [94], while others have determined a greater variability in Se content [95]. The greater variability in Se content in certain studies could also be a consequence of a more efficient Se uptake system in certain plants.

In the research by Lyons et al. [96], the Se content of 665 different varieties of wheat was analyzed and the results indicated that the Se content in the wheat grain ranged from 5 to 720 μg kg^−1^. However, these fluctuations were primarily attributed to the spatial variability of Se in the soil. No substantial variation in Se content in the grain was observed among the examined genotypes. Nevertheless, *Aegilops tauschii* and rye had Se concentrations that were 35% and 45% higher, respectively, compared to other genotypes. The presence of extensive genetic variation in the micronutrient content of wheat grain is crucial for the effectiveness of breeding programs focused on developing genotypes with higher levels of specific micronutrients. Understanding the underlying genetic mechanisms that influence selenium (Se) content is a critical milestone in the genetic biofortification of Se in wheat [82,97].

The identification of QTLs can significantly contribute to breeding programs, and the increasing availability of information on the biochemistry of micronutrient accumulation enables the increasing application of genetic biofortification [89]. QTL analysis is a powerful tool in agronomic research that points to the chromosomal locations of genes suitable for breeding programs. For example, a major QTL from wild two-grain spelt pointing to the chromosomal location of *Gpc-B1*, a gene associated with increased grain protein content and Zn and Fe content, was found and successfully cloned [90].

However, despite its usefulness in understanding the genetic background influencing Se intake, there have been limited studies that have successfully mapped QTLs for Se concentration in wheat grain [82].

Recombinant inbred lines (RILs) and double haploids are mainly used to identify QTLs related to micronutrient concentrations on wheat chromosomes, and some of the most important genetic resources for improving micronutrient content are species related to wheat, which means that the concentration of micronutrients in wheat grain increases in most cases by crossing commercial wheat with its wild relatives that have a greater ability to accumulate micronutrients [98]. The same authors [98] identified a total of 39 QTLs for concentrations of five micronutrients, including Se, using two RIL populations obtained from crosses between SHW-L1 (synthetic hexaploid wheat) and the Chuanmai 32 genotype, and Chuanmai 42 and Chuannong 16 (commercial varieties) (Table 2). In the first population, four QTLs were mapped on chromosomes 3D, 4A, 5B, and 7D, explaining 6.4–28.5% of the genetic variability, while in the second population one QTL was mapped on chromosome 4D explaining 35.1% of the genetic variability for wheat grain Se concentration.

To detect QTLs for Se content in plants at the seedling and adult stages in the field trial and hydroponic culture trial, Wang et al. [97] used a set of RILs derived from two Chinese winter wheat varieties (Tainong18 and Linmai6) by the single-seed descent method. In total, the authors mapped sixteen QTLs for six traits related to Se content on eight chromosomes (1B, 2B, 4B, 5A, 5B, 5D, 6A, and 7D), where seven QTLs were identified for four seedling traits and nine QTLs identified were for two adult traits. Each mapped QTL explained between 7.37 and 20.22% of the total variation in Se grain content (Table 2).

Pu et al. [92] mapped 24 QTLs for traits related to Se content on chromosomes 1B, 3D, 5A, 6A, 6B, 6D, and 7D (Table 2) and also recently documented an Se-rich synthetic wheat line. Furthermore, 50% of the QTLs for grain Se concentration in this study were mapped on chromosome 3D, while the QTL located on chromosome 3D (*Qse.sau-3D*) explained the maximum amount (28.38%) of the genetic variation. Particularly, the aforementioned QTL was identified in several tissues across two successive plant growth cycles in this investigation, and the linked QTLs were consistently expressed throughout the growth period under Se-deficient conditions. Due to this rationale, the authors emphasize the significant importance of the QTL located on chromosome 3D, which has been discovered to be linked to both root features and Se concentration. Given that plants take up micronutrients from the soil through the root system, the authors hypothesize that this QTL improves plant Se uptake by controlling root morphology and structure or Se availability in the plants. Furthermore, the authors in the same study concluded that synthetic hexaploid lines of wheat, as well as their descendants, could be used as a potential genetic source with an improved ability to absorb micronutrients such as Se because a significantly higher concentration of Se was found in both, compared to cultivated wheat.

In the study by Yan et al. [89], the authors found a total of 15 QTLs on chromosomes 1A, 1B, 2B, 3A, 4B, 5A, 6A, 7A, and 7B, which explained 1.4–18.6% of the variation in Se concentration in wheat grain. A total of seven QTLs were associated with grain Se content (GSeC) and a total of eight QTLs were associated with grain Se yield (GSeY). A mapping population consisted of an RIL derived from a cross between wild emmer wheat and durum wheat cultivar Langdon [89] (Table 2). The effectiveness of the breeding programs focused on creating novel plant genotypes rich in micronutrients requires the presence of significant genetic variation for micronutrients in grains and identifying the associated QTLs can help with such breeding programs. QTL analysis is a potent technique in agricultural studies, providing information about the specific place on a chromosome where genes that are suited for breeding programs are found. QTLs regulating Se concentration in wheat grains have been mapped on chromosomes 1A, 1B, 2B, 3A, 3D, 5A, and 5B. Therefore, QTLs for Se content in wheat grain have been determined in several studies on chromosome 5B and, because the same QTLs explained a large part of the genetic variation for Se content, it can be concluded that this particular chromosome is one of the most important for Se concentration in wheat grain, requiring greater attention in the future.

#### 4.2.2. Genetic Engineering of Wheat for Increased Uptake and Accumulation of Selenium

Regarding genetic engineering and transgenic approaches, there are two possible approaches in the field of Se biofortification and phytoremediation to develop high-yielding transgenic plants. The first approach is to develop transgenic plants with efficient uptake and accumulation mechanisms in edible parts and grains in Se-deficient soils and the second approach is to develop plants with the ability to tolerate high amounts and accumulate desirable forms of Se in edible plant parts on soils with high concentrations of Se.

Genetic engineering mainly focuses on genetic manipulations for the purpose of reducing selenate to selenite by the enzyme adenosyl triphosphate sulfurylase (APS), converting SeCys to SeMet by the enzyme cystathionine-γ-synthase (CseGS), avoiding the incorporation of SeCys into proteins by the enzyme selenocysteine methyltransferase (SMT), and volatilizing Se [12]. The first two strategies are related to the direct improvement of Se biofortification potential, while the other two are related to Se tolerance and detoxification mechanisms [12]. The gene for SMT from *Astragalus bisulcatus* was successfully introduced into *Arabidopsis thaliana* to induce a stronger expression of Se-methylselenocysteine and γ-glutamylmethylselenocysteine in the shoot, resulting in enhanced Se accumulation. Other studies have also found that it is possible to mutate certain genes related to Se content in *A. thaliana* to improve the efficiency of crop breeding for Se content at the molecular level [40]. For example, the enhanced expression of the Se binding protein (SBP1) gene in *A. thaliana* improved plant tolerance to selenite through a glutathione-dependent mechanism. Similarly, mutations of the cytosolic ascorbate peroxidase-encoding gene *APX1* or enhanced expression of the ethylene response factor ERF96 improved plant Se tolerance and promoted Se accumulation in *A. thaliana* plants. The enhanced expression of the gene encoding the SMT enzyme in tobacco plants enhanced the accumulation of total Se and Met-SeCys. Another potential target gene is the gene encoding the NRT1.1B transporter, a member of the family of peptide transporters in rice involved in nitrate transport, as it has been shown to exhibit SeMet transport activity, and its enhanced expression in rice resulted in the increased accumulation of SeMet in rice grains [35].

Genetic engineering as an additional breeding technique could therefore, in combination with functional genomics technology, significantly contribute to Se biofortification. Scientists agree that genetic engineering could alter the current scenario of Se deficiency and associated malnutrition, but also its toxicity associated with high concentrations. However, the still unknown, under-researched, and potentially harmful effects of genetically modified organisms associated with the disruption of normal gene function in food crops such as wheat could have irreversible consequences for global food security. Likewise, the presence of ethical barriers in genetic engineering is a limitation for the use of this biotechnology as an alternative to traditional biofortification techniques [12].

## 5. Conclusions

The essentiality of Se has been confirmed for all animals and humans, and the lack of this micronutrient can cause serious health issues. The reliance of a large part of the human population on cereals as a primary source of food and energy is the main cause of malnutrition related to this micronutrient in the world. Although Se supplementation can help reduce diseases and disorders associated with the deficiency of this micronutrient, it is impractical for certain reasons. On the other hand, the biofortification of different crops with Se appears to be a much more economical approach to addressing insufficient Se intake in a large part of the human population. Traditional biofortification approaches, including agronomic biofortification and conventional breeding biofortification, cannot serve the purpose due to various limitations. Genetic engineering, although facing various obstacles regarding the impact of genetically modified organisms on food safety and ethical barriers, appears to be a promising approach for solving the problem of Se deficiency in a large part of the world. Given that it has been proven that the increase in wheat yields in recent years has resulted in a decrease in Se content in wheat grain, there is an increased need for breeding wheat genotypes with improved Se intake. Although significant efforts have been made to study genetic variation for Se content in wheat grain, such research studies are still relatively few and require much greater attention.

## Figures and Tables

**Table 1:** Recommended dietary allowances (RDAs) for selenium (μg day^−1^) [22].

Age Class	Females	Males	Pregnancy	Lactation
<6 months	15	15		
7–12 months	20	20		
1–3 years	20	20		
4–8 years	30	30		
9–13 years	40	40		
14–18 years	55	55	60	70
19–50 years	55	55	60	70
51–70 years	55	55		
>71 years	55	55		

**Table 2:** Overview of references for identification of QTLs for selenium (Se) content in tetraploid and hexaploid wheat.

Reference	Cross	Mapping Population	No. of QTLs for Se Content
	Hexaploid wheat		
[98]	SHW-L1 × Chuanmai 32	171 RILs	39
	Chuanmai 42 × Chuannong 16	127 RILs	
[97]	Tianong 18 × Limma i6	184 RILs	16
[92]	SHW-L1 × Chuanmai 32	171 RILs	24
	Tetraploid wheat		
[89]	LDN × G18-16	152 RILs	15

RIL—recombinant inbred line.

## Data Availability

Data availability is not applicable.
